# Characterization of Coal Fly Ash by Electrical Impedance Spectroscopy, Magnetic Susceptibility and Mössbauer Phase Composition

**DOI:** 10.3390/ma19194232

**Published:** 2026-10-06

**Authors:** Zdzisław Adamczyk, Joanna Komorek, Barbara Solecka, Grzegorz Tytko, Sylwia Dytłow, Andrzej Nowrot, Mariola Kądziołka-Gaweł

**Affiliations:** 1 Department of Applied Geology, Silesian University of Technology, Akademicka 2, 44-100 Gliwice, Poland; zdzislaw.adamczyk@polsl.pl; 2 Department of Electrical Engineering and Automation in Industry, Silesian University of Technology, Akademicka 2, 44-100 Gliwice, Poland; joanna.komorek@polsl.pl (J.K.); andrzej.nowrot@polsl.pl (A.N.); 3 Institute of Physics, Centre for Science and Education, Division of Applied Physics, Silesian University of Technology, Konarskiego 22B, 44-100 Gliwice, Poland; 4 Faculty of Automatic Control, Electronic and Computer Science, Silesian University of Technology, Akademicka 16, 44-100 Gliwice, Poland; grzegorz.tytko@polsl.pl; 5 Institute of Geophysics, Polish Academy of Sciences, Księcia Janusza 64, 01-452 Warsaw, Poland; skdytlow@igf.edu.pl; 6 Institute of Physics, University of Silesia, 75 Pułku Piechoty 1, 41-500 Chorzow, Poland; mariola.kadziolka-gawel@us.edu.pl

**Keywords:** fly ash, electrical impedance spectroscopy, Forster analysis, ferrimagnetic phases, coil impedance, Mössbauer spectroscopy

## Abstract

Fly ashes contain iron-bearing phases with diverse magnetic properties, and their rapid identification may support assessment of their potential for raw-material recovery. The aim of this study is to develop a non-destructive method for characterizing ashes produced from lignite and hard coal combustion. The novel approach integrates electrical impedance spectroscopy (EIS) with magnetic susceptibility measurements and Mössbauer spectroscopy. The investigations were carried out on ashes collected from storage silos and electrostatic precipitators located at power plants. Forster-type impedance characteristics enabled differentiation of the ashes both across a broad frequency range and at a single frequency of 131.8 kHz. The share of spinels among the magnetically ordered phases was very strongly correlated with the real component of impedance and magnetic susceptibility (*r_s_* = 0.964). The results indicate that impedance spectroscopy can be used for a rapid, non-destructive assessment of the proportion of ferrimagnetic phases and the ash suitability for beneficiation.

## 1. Introduction

**Figure 1:**
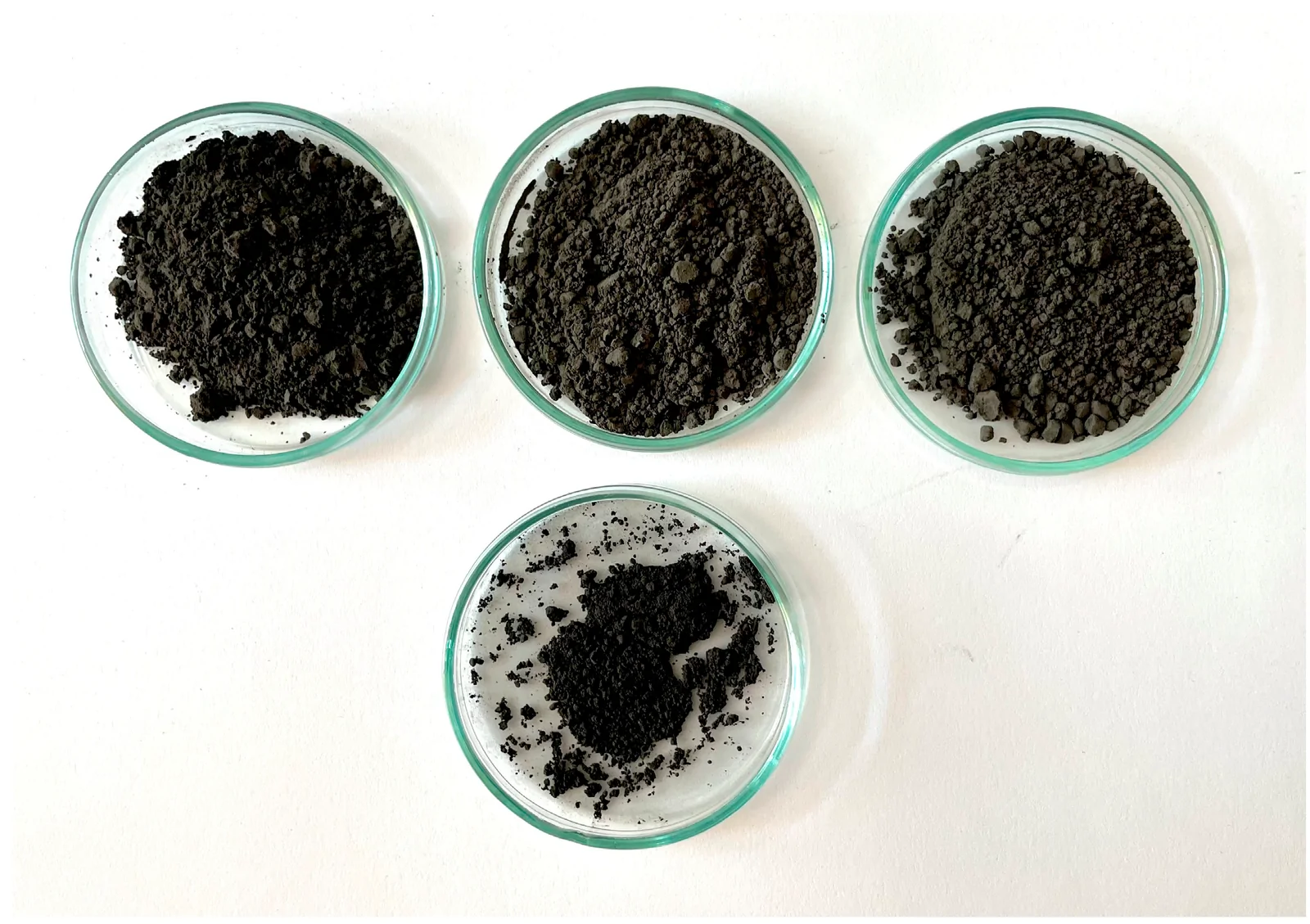
Fly ash produced from the combustion of coal.

Fly ash produced from the combustion of lignite and hard coal is an important by-product of the energy sector and is increasingly used as a secondary raw material in construction, land reclamation, and the recovery of critical raw materials (Figure 1). Its technological applicability is determined primarily by its composition and electromagnetic properties, which are largely related to the presence of iron-bearing phases formed during combustion [1,2,3].

Mineral transformations occurring during combustion lead to the formation of magnetite (Fe_3_O_4_), maghemite (γ-Fe_2_O_3_), hematite (α-Fe_2_O_3_), and other spinel iron-bearing phases. Their type, content, composition, and distribution affect the magnetic and electrical properties of the ash, the efficiency of magnetic separation, and its potential for further use. Identification of Fe phases is therefore important for ash characterization and assessment of its application potential.

Mössbauer spectroscopy makes it possible to determine iron oxidation states and coordination environments and to identify magnetite, hematite, and other iron phases. Magnetic susceptibility measurements allow for a rapid assessment of the fraction of ferrimagnetic phases and are used both in fly ash studies and in environmental pollution monitoring [4,5,6,7]. However, these methods primarily characterize the magnetic response of the material without directly determining the influence of electrical conductivity on its electromagnetic properties. This limitation can be overcome by impedance spectroscopy (IS). Such measurement methods are widely used to characterize the electrical properties of ceramics, composites, and functional materials using electrodes in contact with the sample [8,9,10,11]. In the present study, electrical impedance spectroscopy (EIS) was applied [12,13,14,15]. In this contactless technique, the sample placed inside a coil acts as an effective core. The presence of the material changes the coil impedance, which depends on its magnetic permeability and electrical conductivity. This feature enables rapid, non-destructive characterization of bulk materials without the use of electrodes.

The results were interpreted according to the Förster concept, using effective magnetic permeability and normalized functions describing changes in the impedance components. This approach enables better discrimination of materials with similar electromagnetic responses than classical Nyquist plot analysis. The application of the Förster approach enables a more detailed analysis of the electromagnetic response of the investigated material than the conventional Nyquist representation, particularly when identifying subtle differences between samples with similar electromagnetic properties. In Nyquist plots, the relationship between the real and imaginary components of impedance is not presented directly as a function of frequency. In contrast, analysis of the coil parameters as a function of frequency makes it possible to identify the range in which the largest variation in its response occurs. Furthermore, using the empty-coil characteristics as a reference, together with normalized changes in resistance and inductance, reduces the influence of the intrinsic parameters of the measurement system.

This approach is particularly advantageous for investigating powdered magnetic materials because it enables identification of the frequency range with the greatest sensitivity to changes in effective magnetic permeability and electromagnetic losses associated with the presence of magnetic phases, such as magnetite Fe_3_O_4_.

It is particularly useful for fly ashes, whose impedance characteristics depend on the content and type of ferrimagnetic spinels. The proposed method belongs to the developing group of contactless inductive techniques and AC magnetometry based on air coils [16,17,18,19,20].

To date, contactless impedance methods have been used mainly for the study of metal powders, conductive materials, and AC magnetometry [16,17,18,19,20]. The novelty of this work lies in the first application of electrical impedance spectroscopy with Förster analysis to characterize fly ash generated from lignite and hard coal combustion. The raw data were correlated with results obtained by Mössbauer spectroscopy and magnetic susceptibility measurements. The analysis made it possible to determine relationships between impedance response parameters, the magnetic properties of the ashes, and the content of iron-bearing phases. The study confirmed the possibility of using impedance parameters as rapid indicators of ferrimagnetic phase content. In addition, it demonstrated the suitability of the ashes for magnetic beneficiation, recovery of raw material components, and material applications.

## 2. Research Methods

### 2.1. Research Material

Fly ash samples were collected from two Polish power plants designated B and L. Power plant B burns low-rank lignite B, a metalignite with an average to high ash content. Power plant L burns ortho-bituminous hard coal, medium-rank C, with high vitrinite content and moderate ash content. The samples used in the study came from the storage silo of power plant L (LMOS) and from three generating units (1, 2, and 3) at power plant B. These included both storage-silo ashes (BL1, BL2, and BL3) and electrofilter ashes (BF1, BF2, and BF3) collected from installations associated with those units. The sample designations used in the subsequent parts of the study are shown in Table 1.

Samples with a mass of approximately 100 g were subjected to magnetic separation. The separation was performed manually using a dry method and a neodymium magnet protected with foil. The material under study was spread in a layer 1–3 mm thick, and the magnet was moved above its surface at a distance of approximately 5 mm. Attracted grains were removed progressively, and the procedure was repeated until no further attraction of material by the magnet was observed. Depending on the sample, this required 5 to 15 repetitions and made it possible to separate the magnetic and non-magnetic fractions.

### 2.2. Phase Component Identification

The identification of iron-containing phases was carried out using an integrated Mössbauer spectroscopy measurement system (designed by Wacław Musiał and Jacek Marzec, Cracow, Poland). The ^57^Fe Mössbauer spectra were recorded at room temperature using a spectrometer operating in the constant-acceleration mode. The measurement setup had a transmission geometry and included a ^57^Co:Rh radiation source, an absorber, a detector, and a multichannel analyzer using 1024 channels before spectrum folding. The velocity scale was calibrated using a 30 µm α-Fe foil. Numerical analysis and fitting of the recorded spectra were performed using the MossWinn4.0i software. The isomer shifts are given relative to α-Fe at room temperature. Due to the low intensity of resonant absorption, the acquisition time for individual spectra ranged from 48 to 350 h.

### 2.3. Electrical Impedance Spectroscopy

**Figure 2:**
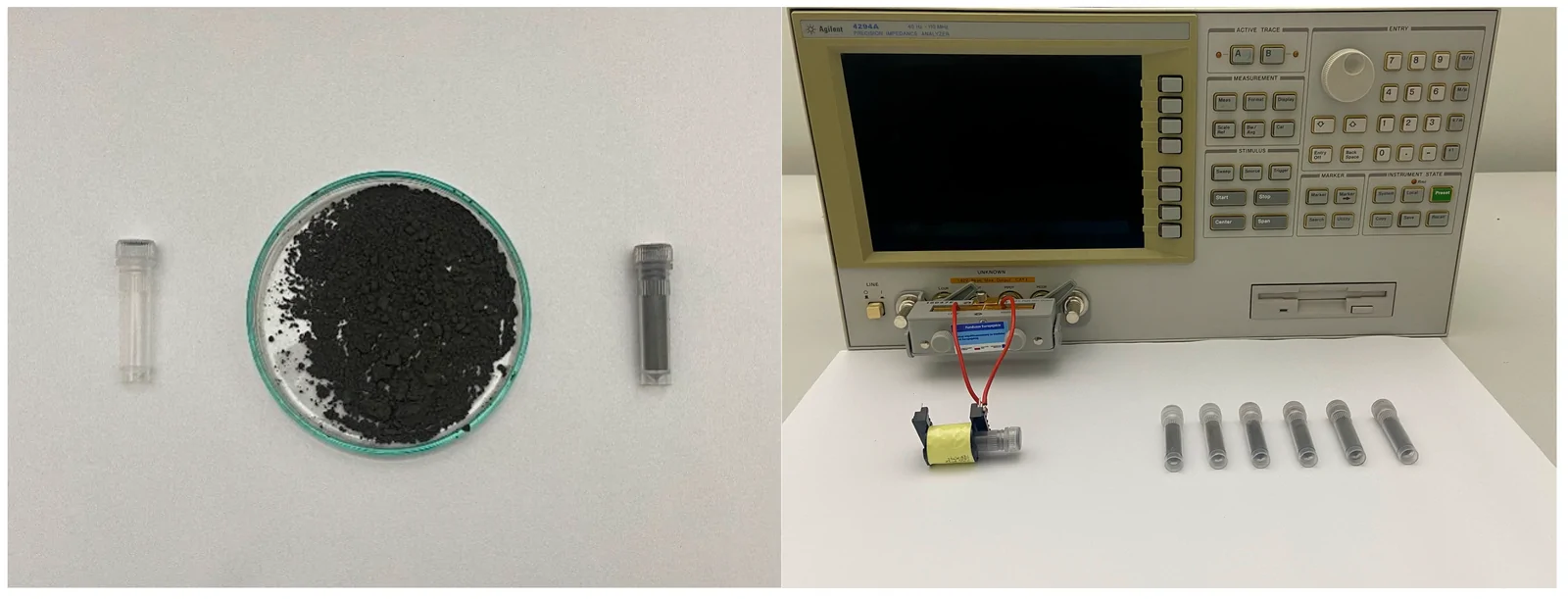
**Left** photograph: an empty ampoule, fly ash, and an ampoule containing fly ash. **Right** photograph: the measurement setup comprising an Agilent 4294A precision impedance analyzer, a measurement coil, and ampoules.

In studies of fly ash, an electrical impedance spectroscopy method was used with a measuring coil in which the sample served as the magnetic core. In contrast to electrochemical impedance spectroscopy [8,9,10,11], where the measurement signal passes directly through the sample using electrodes, the method applied here is contactless. This solution makes it possible to assess simultaneously the electrical and magnetic properties of bulk materials, in particular the content of ferromagnetic and conducting phases [14,15,16,17,18,19,20]. The measurement is based on recording the complex impedance of the coil while sweeping a wide frequency range. The impedance analyzer generates a sinusoidal voltage signal at a specified frequency and measures the current response of the system under study, determining the magnitude of the impedance and the phase shift angle between voltage and current. On the basis of these parameters, the real and imaginary components of the complex impedance are calculated, forming the basis for further spectral analysis and interpretation of electromagnetic processes occurring in the material under study. The frequency response of the impedance is determined by the electromagnetic properties of the sample, in particular its electrical conductivity, relative magnetic permeability, microstructure, and the degree of compaction and porosity of the material [14,15,16,17,18,19,20]. Fly ash samples were poured into a cylindrical ampoule made of non-magnetic material, which was then placed inside the measuring coil (Figure 2). To ensure reproducibility of the results, all samples had the same volume and the same degree of filling of the ampoule. Before each measurement series, the system was calibrated by determining the frequency response of the empty coil. The recorded impedance spectrum of the empty coil served as a reference for further analysis of changes induced by the presence of the material under study. A similar approach is currently used in modern AC magnetometry systems and impedance sensors based on air coils [16,17,18,19,20]. After determining the impedance of the empty coil, *Z*_0_ = *R*_0_ + *jωL*_0_, the sample was introduced into its interior. The measured coil impedance, *Z* = *R* + *jωL*, changed as a result of the interaction between the magnetic field and the electrically conductive ash particles. The analysis covered changes in the impedance components as a function of frequency, reflecting changes in the magnetic permeability and electrical conductivity of the material under study. The obtained results were interpreted according to the method proposed by F. Förster, using the concept of effective magnetic permeability of the material.

In this model, the normalized coil impedance is described by the relationship:

(1) $$\frac{Z}{\omega L_{0}}\approx j\left(1-\eta +\eta \mu_{r}\mu_{\mathrm{eff}}\right),$$

where *η* denotes the coil filling factor by the sample, *μ_r_* is the relative magnetic permeability of the material, and *μ_eff_* is the effective magnetic permeability. This approach currently underlies many methods for determining the electromagnetic properties of materials using measuring coils and impedance spectroscopy [12,13,14,15].

To increase the sensitivity of the analysis, the functions proposed by Förster were used:

(2) $$h_{1}\left(\omega \right)=\frac{R-R_{0}}{\omega L_{0}}=-\eta \mu_{r}Im\left\{\mu_{\mathrm{eff}}\right\},$$

(3) h2ω=ωLωL0=1−η+ημrReμeff,

The function describes changes in the real component of impedance, mainly associated with the magnetic losses of the material, whereas the function corresponds to changes in the inductive component related to the real part of the effective magnetic permeability. The use of these functions makes it possible to distinguish materials with similar impedance characteristics much more accurately than classical Nyquist plot analysis, as confirmed in more recent applications of measuring coils and contactless EIS [14,15,16,17,18,19,20]. This is particularly important for materials originating from lignite or hard coal combustion, such as fly ash, bottom ash, or mixtures containing iron oxides, whose magnetic properties differ only slightly but still affect the course of the impedance spectrum.

The obtained frequency characteristics of the functions and constitute the basis for identifying the electromagnetic properties of the investigated bulk materials. Analysis of changes in the positions of maxima and minima, as well as the overall course of both functions, makes it possible to assess the content of magnetic phases, the degree of electrical conductivity, and microstructural changes in waste materials originating from lignite combustion. Due to the contactless nature of the measurement, the method can be used for rapid and non-destructive assessment of bulk materials without the need for electrodes or special sample surface preparation.

### 2.4. Magnetic Susceptibility χ and Frequency-Dependent Magnetic Susceptibility χ_fd_% Measurements

**Figure 3:**
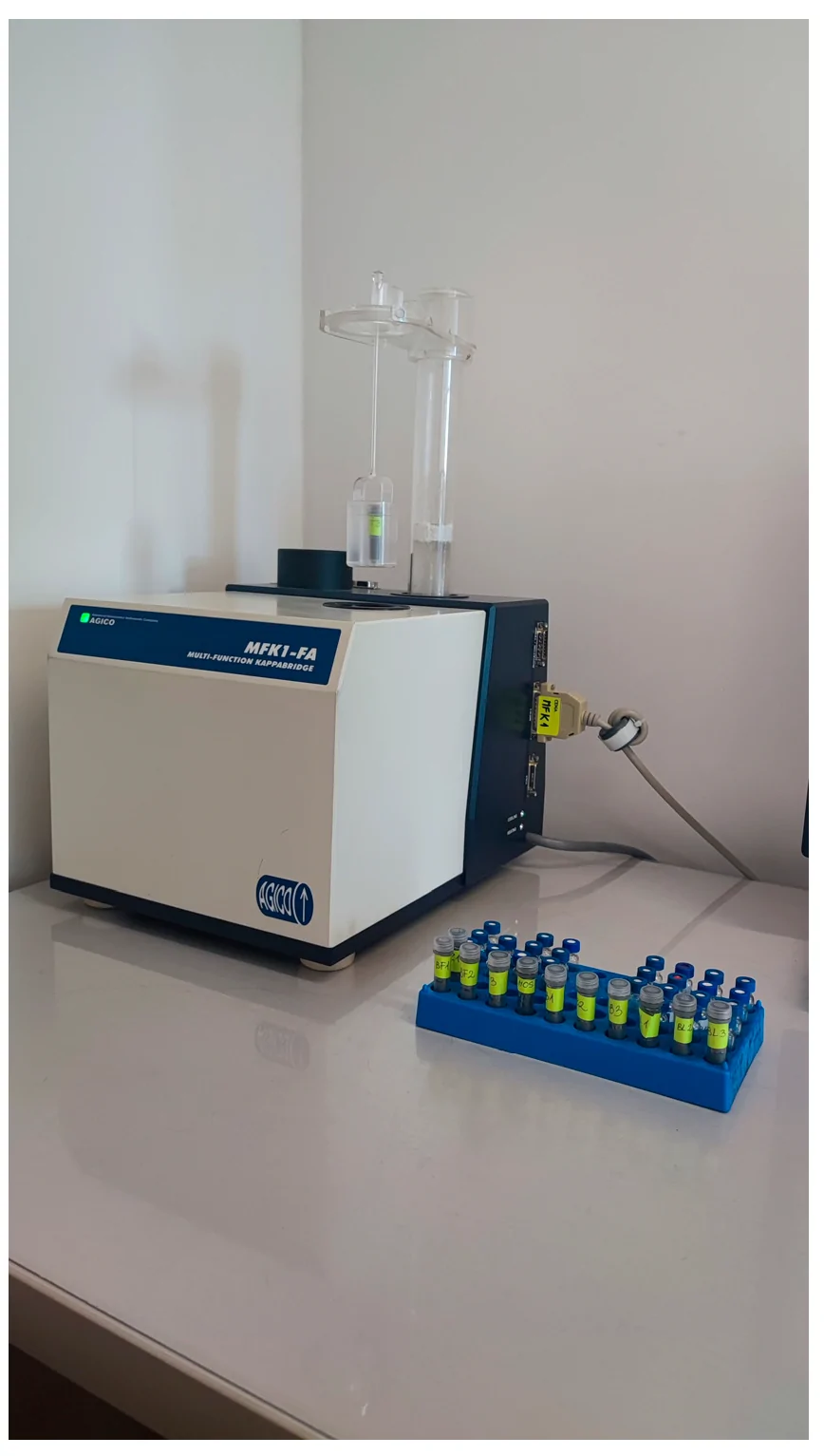
Experimental setup for magnetic susceptibility measurement using the MFK1-FA Multi-Frequency Kappabridge (AGICO) alongside prepared environmental samples in non-magnetic vials.

The magnetic susceptibility *χ* serves as a key indicator of a material’s magnetization capacity when subjected to an external magnetic field [21]. The magnitude of this parameter is determined by the mineralogical makeup, the concentration of magnetic minerals, and the abundance of fine-grained particles [22]. For this research, specimens were analyzed in standard plastic vials. Measurements were conducted employing a multifunctional MFK1-FA Kappabridge (AGICO, Brno, Czech Republic) at dual frequencies: a low frequency of 976 Hz and a high frequency of 15,616 Hz (Figure 3). The experimental setup utilized a magnetic field strength of 200 A/m and achieved a sensitivity of 2 × 10^−8^ SI. Selecting this particular field intensity is advantageous for emphasizing the signal from ferromagnetic components, which saturate at lower fields, while simultaneously minimizing the contribution from paramagnetic minerals [22,23]. To determine the mass-specific magnetic susceptibility *χ* (m^3^/kg), the following Equation (4) was applied:

(4) $$\chi =\frac{K\times V_{0}}{m},$$

where *χ* is the mass-specific magnetic susceptibility (m^3^ kg^−1^), *κ* is the dimensionless volume magnetic susceptibility, *V*_0_ is the calibration constant volume of 1 × 10^−5^ m^3^ (10 cm^3^) for the MFK1-FA kappabridge, and m is the sample mass (kg).

Mass-specific magnetic susceptibility *χ* (m^3^ kg^−1^) was determined using the MFK1-FA Kappabridge operating in mass-specific mode. We entered a nominal mass of 1 g in the software and recalculated the values to the actual sample mass when necessary. Normalization to mass minimizes the influence of variable bulk density, porosity and packing density of the ash, thereby enabling a more reliable comparison of the abundance and properties of magnetic phases among the studied samples.

To evaluate the relative variation in susceptibility across the two frequencies, the frequency-dependent magnetic susceptibility *χ_fd_*% was calculated. Initially, the absolute difference was established as *χ_fd_* = *χ_lf_* − *χ_hf_*, which was then expressed as a percentage according to Equation (5):

(5) $$\chi_{\mathrm{fd}}\%=\frac{\left(\chi_{\mathrm{lf}}-\chi_{\mathrm{hf}}\right)}{\chi_{\mathrm{lf}}}\times 100,$$

where *χ_lf_*% and *χ_hf_*% represent the magnetic susceptibility values recorded at low and high frequencies, respectively [22,24].

## 3. Results and Discussion

The mass yield of the magnetic fraction showed considerable variability among the studied samples, ranging from 17.8 wt.% for sample M-BL2 to 54.5 wt.% for M-BF2 (Table 2). The M-BF samples were characterized by the highest yield of the magnetic fraction, from 30.3 to 54.5 wt.%, with an average of 43.0 wt.%. In contrast, the M-BL samples showed a lower and more uniform yield, in the range of 17.8–20.7 wt.%, with an average of 19.0 wt.%. The collected samples were subjected to magnetic separation using a neodymium magnet, resulting in two fractions: magnetic and non-magnetic. The remainder of the manuscript presents results obtained exclusively for the magnetic fraction. Thus, the average yield of the magnetic fraction from the M-BF samples was about 2.3 times higher than that from the M-BL samples. For the M-LMOS sample, the yield was 21.1 wt.% and was similar to the values obtained for the M-BL samples.

The results indicate significant differences in the content of components responsive to the applied magnetic field in the studied materials. However, it should be emphasized that the mass yield of the magnetic fraction does not directly determine the degree of iron enrichment. Its assessment requires consideration of the chemical and mineralogical composition of the separated magnetic fractions.

### 3.1. Mössbauer Spectroscopy

**Figure 4:**
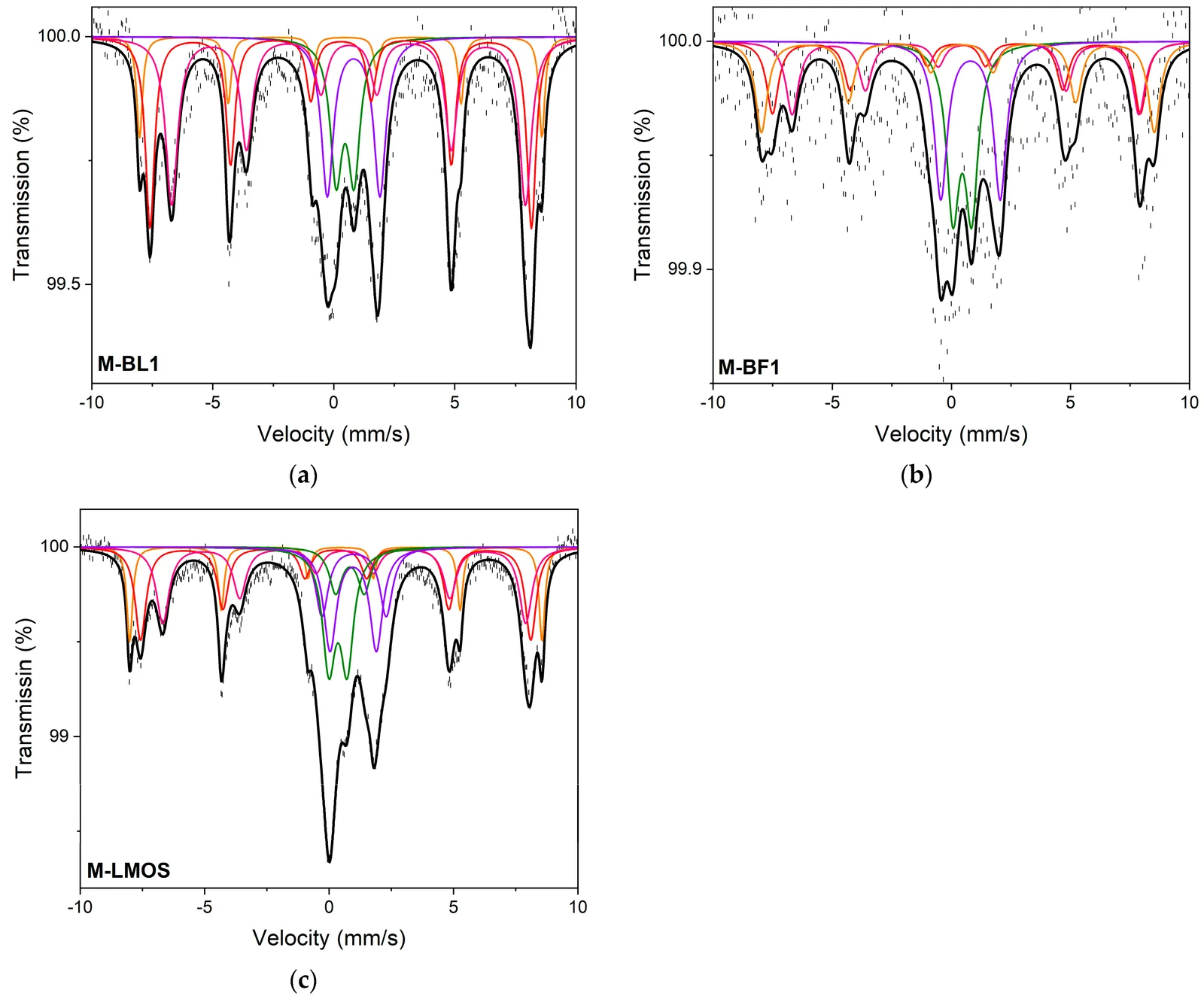
Representative Mössbauer spectra together with fitted components of the magnetic fractions of (**a**) M-BL1, (**b**) M-BF1 and (**c**) M-LMOS samples; where vertical lines—experimental points, black lines—fit, orange lines—Fe_2_O_3_; red lines—tet-Fe_3_O_4_, pink lines—Oct-Fe_3_O_4_, olive lines—Fe^3+^ in aluminosilicates/silicates, violet lines—Fe^2+^ in aluminosilicates/silicates.

The Mössbauer spectra of the magnetic fractions of the studied samples include three magnetically ordered components: a hematite sextet and two sextets assigned to iron in the tetrahedral (A) and octahedral (B) positions of magnetite, in accordance with descriptions of iron phases in fly ash reported in the literature [24,25,26,27]. The paramagnetic components, described by Fe^3+^ and Fe^2+^ doublets, were mainly associated with iron dispersed in the aluminosilicate glass. Representative spectra are shown in Figure 4, and the fitting parameters are summarized in Table 3 and Table 4.

**Figure 5:**
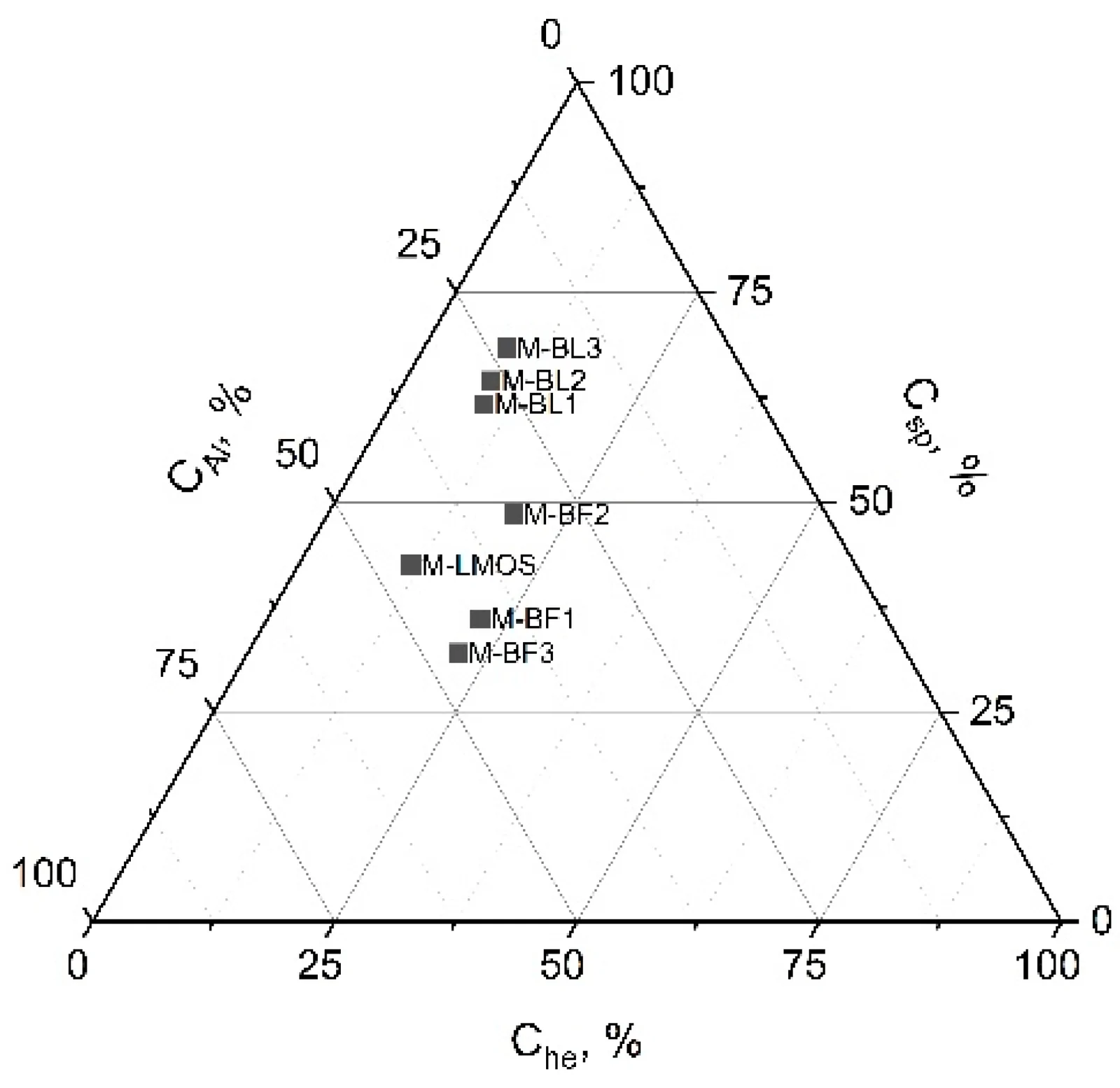
The proportion of iron in magnetic and ferromagnetic phases.

The highest proportion of iron associated with spinel phases, *C_sp_*, was found in the M-BL1–M-BL3 samples, where it accounted for 61.7–68.4% of the relative spectral area. Hematite, *C_he_*, accounted for 8.6–9.6%, so the total share of iron in the magnetically ordered components, *C_mag_*, ranged from 71.3 to 76.9%. Spinels made up 86.5–88.9% of the magnetically ordered components, *C_sp_*_/*mag*_ (Figure 5, Table 5). The dominance of magnetite and the minor contribution of hematite are typical for the magnetic fractions of fly ash; maghemite may also be present, formed among other processes by the oxidation of magnetite [24,25,26,27,28,29]. The Fe^2+^ and Fe^3+^ doublets, *C_Al_*, were assigned 23.1–28.7% of the iron in the M-BL series. Their presence in the magnetic concentrate can be explained by the co-separation of aluminosilicate glass, mineral intergrowths, and unburned organic matter with the grains of iron-bearing phases. Compared with the M-BL series, the M-BF1–M-BF3 fractions contained a higher proportion of iron associated with hematite, *C_he_* (19.1–22.0%), and a lower proportion of spinels, *C_sp_* (32.0–48.6%). At the same time, the share of the paramagnetic *C_Al_* components was higher, at 32.3–46.2%, whereas spinels accounted for 59.6–71.7% of the magnetically ordered components, *C_sp_*_/*mag*_ (Figure 5, Table 5).

The differences between the M-BF and M-BL series may be related to a greater degree of iron oxidation in the finer ash fraction, a higher proportion of intergrowths between non-magnetic components and iron-bearing grains, and the limited selectivity of dry separation, which is consistent with observations for fine fractions from electrofilters and magnetic concentrates [30,31,32,33,34]. The increased share of iron among the paramagnetic Fe^2+^ and Fe^3+^ *C_Al_* components (33.2–46.2%) is also consistent with the presence of intergrowths and aggregates in which magnetite may occur on the surface of, or within, aluminosilicate spheres, making selective phase separation difficult.

Studies by Szumiata et al. [35] confirm that finer electrofilter fractions are characterized by a higher ratio of iron incorporated in aluminosilicate (glassy) phases to iron present in spinel phases, and that finer grains consistently contain more glassy iron-bearing material.

Combustion conditions significantly affect iron speciation by altering the relative proportions of glass, spinels, magnetite, maghemite, and hematite, without necessarily forming new phases [36,37]. In fly ash, an increase in temperature favored the formation of magnetite at the expense of hematite, maghemite, hercynite, and iron bound in mullite, and its content was proposed as an indicator of combustion temperature [37].

In the M-LMOS sample, 42.5% of the iron was assigned to the spinel phases, *C_sp_*, 11.6% to hematite, *C_he_*, and 45.9% to the paramagnetic Fe^2+^ and Fe^3+^ components, *C_Al_*. The total share of iron in the magnetically ordered components, *C_mag_*, was 54.1%, of which 78.5% belonged to the spinels, *C_sp_*_/*mag*_ (Figure 5, Table 5). The high contribution of doublets confirms that magnetic separation concentrates not only individual magnetite grains, but also intergrowths and aluminosilicate spheres containing dispersed iron.

### 3.2. Electrical Impedance Spectroscopy (EIS)

**Figure 6:**
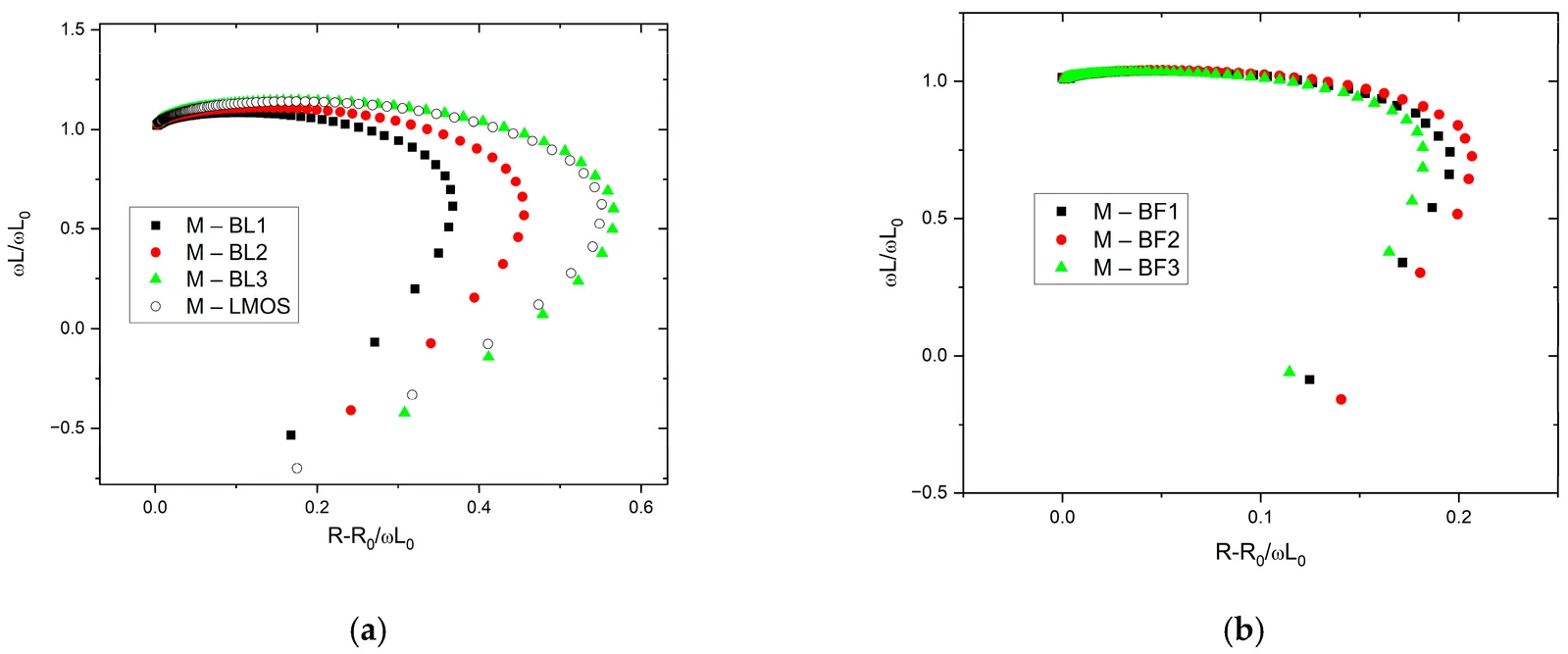
Normalized impedance characteristics of fly ash originating from a lignite-fired power plant: (**a**) M-BL1–M-BL3 ashes and a comparative sample from a hard coal-fired power plant (M-LMOS), (**b**) M-BF1–M-BF3 ashes.

The normalized impedance diagrams (Förster characteristics) determined for fly ash originating from a lignite-fired power plant reveal the relationship between the normalized imaginary component $\frac{\omega L}{{\omega L}_{0}}$ and the normalized real component $\frac{R\;-\;R_{0}}{\omega L_{0}}$, making it possible to compare the impedance changes in individual samples across the entire analyzed frequency range (Figure 6). This type of data presentation is widely used in electrical impedance spectroscopy to compare the electrical response of materials to variable-frequency excitation.

Due to the complex, multiphase nature of fly ash, no absolute calibration of the impedance parameters against a single magnetic standard was performed. The investigated material may contain coexisting Fe-bearing phases characterized by different magnetic permeabilities, electrical conductivities, and frequency responses. The use of Fe_3_O_4_ as the sole reference material could be unrepresentative and might lead to an erroneous conversion of the impedance response into the mass fraction of the magnetic component. Therefore, the EIS results were treated as relative indicators of the effective electromagnetic properties of the investigated material volume. From the perspective of analyzing the results obtained for M-BL1–M-BL3 and the comparative sample M-LMOS, the most important feature is the frequency range of maximum sensitivity of the measuring coil, in which the normalized impedance characteristics show the greatest mutual divergence, providing the highest ability to distinguish the materials under study (Figure 6a). In this region, the curves for the individual samples separate clearly, as seen in both the normalized real and imaginary components of impedance. For the M-BL samples, the differences cover a wide range of changes in the normalized real component, from about 0.37 for M-BL1 to about 0.58 for the comparative sample M-LMOS. In the case of the M-BF samples, the separation of the characteristics is smaller, but still clearly visible; the normalized real component reaches maximum values of approximately 0.17–0.21. The Förster characteristics for samples M-BF1– M-BF3 have a similar shape, but cover a much narrower range of changes in the normalized real component (Figure 6b), while the normalized imaginary component reaches maximum values of about 1.03–1.04.

For samples M-BL1–M-BL3 and the comparative sample M-LMOS (Figure 6a), a similar initial course of the characteristics is observed, with *L*/*L*_0_ values close to unity. As the frequency changes, the characteristics shift towards higher values of (*R* − *R*_0_)/*L*_0_, accompanied by a decrease in *L*/*L*_0_. However, the extent of this shift differs among the individual samples. M-BL1 reaches horizontal-coordinate values of approximately 0.37, M-BL2 approximately 0.45, and M-BL3 approximately 0.55–0.56. The widest range of variation, of approximately 0.58, is observed for the comparative sample M-LMOS. This variation in curve positions indicates differences in the intensity of interactions between the individual materials and the electromagnetic field of the coil.

The characteristics of M-BF1–M-BF3 (Figure 6b) exhibit a similar overall shape; however, they cover a substantially narrower range of variation along the horizontal coordinate, approximately 0–0.21. The highest (*R* − *R*_0_)/*L*_0_ values are obtained for M-BF1 and M-BF2, whereas M-BF3 shows a slightly smaller shift towards the horizontal axis. At the same time, the maximum *L*/*L*_0_ values for all M-BF samples are very similar, ranging from approximately 1.03 to 1.04. This indicates that the differences among samples in this group are more evident in changes in the real component than in the imaginary component of impedance.

Comparison of the two groups indicates that the characteristics of M-BL1–M-BL3 and M-LMOS show substantially greater variation than those of M-BF1–M-BF3. The greatest separation of the curves occurs in the range corresponding to increased sensitivity of the measurement coil. In this region, minor differences in the electromagnetic properties of the samples lead to distinct differences in the positions of the Förster characteristics. From the perspective of sample identification and differentiation, this is a particularly important part of the characteristics because it provides the greatest resolving power of the method.

In both plots, the greatest separation of the characteristics occurs in the frequency range corresponding to the maximum sensitivity of the measuring system. In this region, the largest differences in the normalized impedance components between the individual samples are observed, which makes it possible to compare them unambiguously. In the initial and final ranges of the characteristics, the differences between the curves are clearly smaller.

The electromagnetic nature of EIS enables the application of Förster analysis to compare the properties of different material samples. According to the approach developed by Förster, the electrical properties of the investigated material are represented by its electrical conductivity, *σ*, whereas its magnetic properties are represented by its magnetic permeability, *μ*. These parameters are compared by interpreting the positions of the curves in impedance diagrams. A prerequisite for the correct interpretation of these plots is the use of measurement data obtained at the same operating frequency of the coil. Electrical conductivity is determined by the value of *ωL*/*ωL*_0_ shown on the vertical axis. For this purpose, the lower portion of each plot is the most convenient region to consider.

The higher the electrical conductivity, the lower the value of *ωL*/*ωL*_0_; consequently, the points on the curve shift downward. Thus, based on Figure 6a, the *σ* values for M-BL1, M-BL2, and M-LMOS are similar but lower than that for M-BL3. In Figure 6b, sample M-BF2 exhibited the highest electrical conductivity, whereas M-BF3 exhibited the lowest.

In fly ash studies, electrical conductivity provides valuable information on the total content of electrically conductive constituents, including heavy metals and magnetic particles. Conventional approaches to determining *σ*, such as four-point and six-point probe methods or conductometric measurements, are rarely used for granular materials because they are associated with substantial uncertainty or require complex sample preparation, for example, by sintering. Although Förster analysis based on EIS measurements does not provide numerical values of *σ*, it enables the electrical conductivities of granular materials to be compared. The magnetic properties of the investigated material can be interpreted from impedance diagrams in a similar manner.

The higher the magnetic permeability of a material, the higher the value of *ωL*/*ωL*_0_ on the vertical axis. These observations are most conveniently made in the upper portion of the plot, corresponding to low operating frequencies of the probe. In Figure 6a, the highest *μ* values were observed for M-BL3 and M-LMOS, whereas the lowest value was found for M-BL1. The results shown in Figure 6b indicate that the magnetic permeability values of all three samples were similar. The highest *μ* was obtained for M-BF2, with only slightly lower values for M-BF1 and M-BF3.

At the same time, the *ωL*/*ωL*_0_ values obtained for the samples shown in Figure 6a were higher than those in Figure 6b, indicating stronger magnetic properties. The ability to compare granular materials on the basis of magnetic permeability, provided by Förster analysis, is particularly valuable because determining *μ* across the full operating-frequency range of the probe is complex and time-consuming.

The observed differences in the impedance characteristics can be related both to the total iron content and, more importantly, to the proportion of magnetic phases and the presence of amorphous matter. However, total Fe concentration is not a direct indicator of a sample’s magnetic properties, as iron may occur both in magnetic phases, such as magnetite Fe_3_O_4_, and in weakly magnetic or amorphous phases. An increased contribution of the magnetic fraction leads to higher effective magnetic permeability and electromagnetic losses, which are reflected in changes in the coil inductance and resistance and, consequently, in shifts in the normalized Förster characteristics.

The amorphous phase may, in turn, modify the impedance response by affecting the effective electrical conductivity, the structure of interparticle contacts, and the mode of occurrence of iron within the sample matrix. Therefore, relating the impedance characteristics to the contribution of magnetic phases, total Fe content, and the amorphous fraction enables a more reliable identification of the factors responsible for variations in the electromagnetic response of the investigated materials.

**Figure 7:**
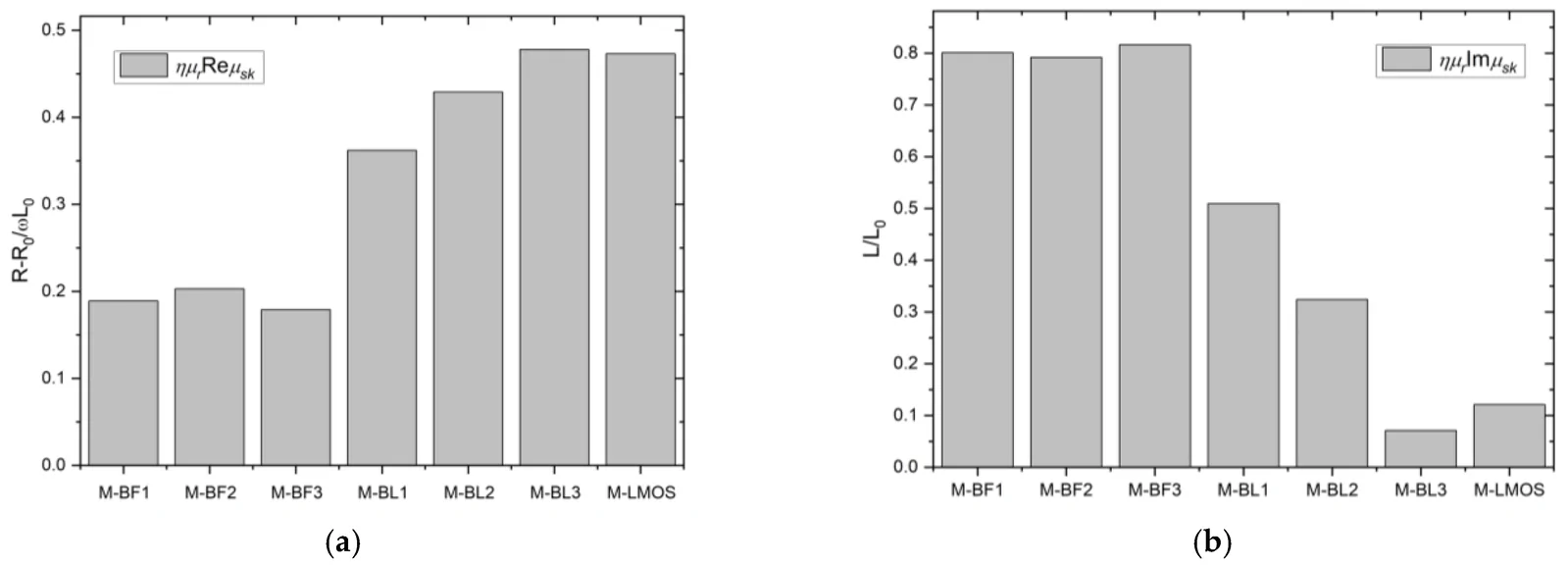
Comparison of the normalized impedance component values determined at a single measurement frequency, *f* = 131.8 kHz, corresponding to the range of maximum sensitivity of the measuring coil: (**a**) normalized real part of impedance, (**b**) normalized imaginary part of impedance for M-BF1–M-BF3 ashes, M-BL1–M-BL3 ashes, and the comparative sample (M-LMOS).

The second pair of plots (Figure 7) presents a comparison of the normalized real and imaginary impedance values determined at one selected measurement frequency, *f* = 131.8 kHz. This frequency was chosen from the range corresponding to the highest sensitivity of the measuring coil, that is, from the region in which changes in the electromagnetic parameters of the material under study cause the greatest changes in the normalized impedance components. In practice, selecting such a frequency point makes it possible to achieve the highest measurement resolution and the best ability to differentiate the materials under study.

The plot showing the normalized real part of impedance $R-\frac{R_{0}}{\omega L_{0}}$ reveals a clear division of the investigated samples into two groups. The samples labeled M-BF1–M-BF3 reach similar values, in the range of 0.18–0.20, which indicates only minor differences between them at the analyzed measurement point. In contrast, the M-BL1–M-BL3 samples show a gradual increase in the real part of impedance, from 0.36 for M-BL1 to 0.47 for M-BL3. The comparative sample M-LMOS reaches a value similar to M-BL3, also at about 0.47. A similar comparison of the normalized imaginary part of impedance shows a different distribution of values. The highest values were recorded for samples M-BF1–M-BF3, for which this parameter is about 0.79–0.81. In the M-BL samples, a gradual decrease is observed, from 0.50 for M-BL1 to 0.07 for M-BL3. The comparative sample M-LMOS reaches a value of 0.12, close to that of M-BL3. Presenting the results for a single, optimally selected frequency complements the analysis of the full impedance characteristics shown in the Förster plots. Choosing a frequency from the range of maximum sensitivity of the measuring coil makes it possible to compare the values of both normalized impedance components directly for all investigated samples, without analyzing the entire frequency spectrum. This type of presentation is commonly used in methods based on normalized impedance functions, because in the region of highest system sensitivity even small changes in the electromagnetic properties of the material cause distinct changes in both the real and imaginary parts of impedance.

### 3.3. Magnetic Susceptibility χ and Frequency-Dependent Magnetic Susceptibility χ_fd_% Measurements

**Figure 8:**
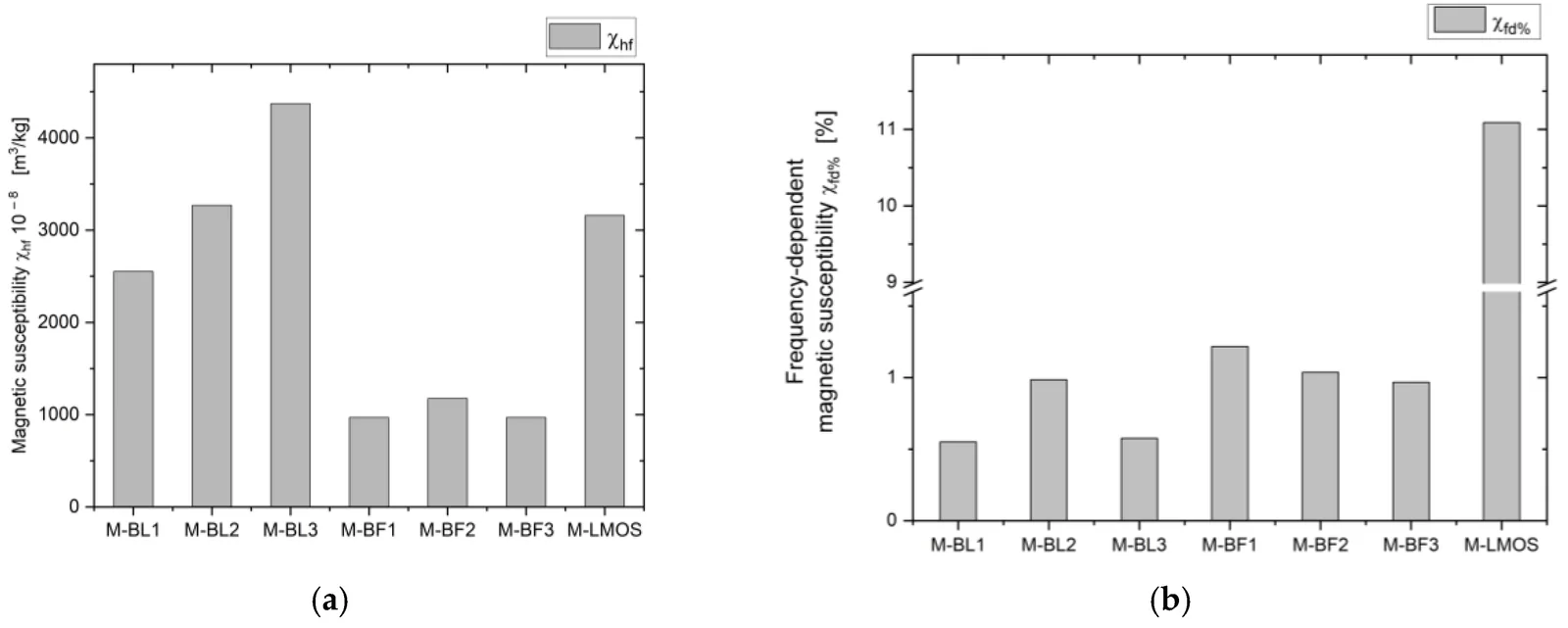
Magnetic susceptibility *χ_lf_* at low frequency (**a**) and frequency-dependent magnetic susceptibility *χ_fd_*% (**b**) of the investigated magnetic fractions from coal combustion by-products. Samples are grouped by power plant and collection site: M-BL = flue gas duct fly ash (Power Plant B), M-BF = electrostatic precipitator fly ash (Power Plant B), M-LMOS = hard-coal bottom ash (Power Plant L).

The statistical analysis of the entire data set (Figure 8, Table 6) reveals a wide range of magnetic properties in the investigated samples. For the whole population, the average low-field mass-specific susceptibility, *χ_lf_*, was 2235.2 ± 1425.8 × 10^−8^ m^3^/kg, with values ranging from a minimum of 978.2 × 10^−8^ m^3^/kg to a maximum of 4396.0 × 10^−8^ m^3^/kg.

Detailed provenance analysis shows that the combustion products of lignite from power plants B and L exhibit significant internal variability. Fly ash from power plant B differed depending on the sampling location: samples from the storage silos (M-BL1–M-BL3, mean *χ_lf_* ≈ 3421.2 × 10^−8^ m^3^/kg) had a markedly higher *χ_lf_* value than samples from the electrofilters (M-BF1–M-BF3, mean *χ_lf_* ≈ 1049.2 × 10^−8^ m^3^/kg). As for frequency-dependent susceptibility, the overall mean *χ_fd_*% was 0.89 ± 0.27%, but this parameter showed a large divergence between fuel types. While samples derived from lignite from power plants B and L consistently showed low values, ranging from 0.55% to 1.22%, the sample derived from hard coal from power plant L (M-LMOS) stood out significantly, reaching a *χ_fd_*% value of 11.09%. This exceptionally high value for hard-coal fly ash, compared with the consistently low values in lignite-derived products, indicates a unique magnetic mineralogy dominated by a substantial contribution of superparamagnetic (SP) grains.

Correlation analysis is one of the fundamental statistical tools used to identify relationships between investigated variables and assess their interdependence. In studies of magnetic properties of materials, it makes it possible to determine which mineral components most strongly control the magnetic response of the samples, and it also supports interpretation of the mineralogical and geochemical processes responsible for shaping their properties.

Spearman rank correlation analysis (Table 7) revealed strong relationships between the mineral composition of the samples and their magnetic properties. The proportion of spinel iron oxides with ferrimagnetic properties shows a very strong positive correlation with the parameters $R\;-\frac{\;R_{0}}{\omega L_{0}}$, *χ_lf_*, and *χ_hf_* (*r_s_* = 0.964), and a negative correlation with hematite content (*r_s_* = −0.964) (Table 7). At the same time, hematite content is strongly negatively correlated with the share of spinel phases (*r_s_* = −0.929), indicating a gradual transformation between these mineral components.

The observed relationships may be interpreted as an effect of the reduction of hematite (α-Fe_2_O_3_) to magnetite (Fe_3_O_4_), occurring under conditions of limited oxygen availability or in the presence of reducing agents. This process is associated with partial reduction of Fe^3+^ to Fe^2+^ and a restructuring of the oxide lattice, leading to the formation of magnetite with a spinel structure and ferrimagnetic properties. As a result, a decrease in hematite content and an increase in the proportion of spinel phases cause an increase in the magnetic parameter values, which is confirmed by the obtained correlation coefficients. The strong positive relationship between the share of ferrite spinel and the *χ_lf_* and *χ_hf_* parameters indicates that an increase in the amount of magnetite/maghemite is the main factor controlling the rise in the magnetic response of the studied samples. By contrast, the lack of clear relationships between *χ_fd_* and the other variables (∣*r_s_*∣ < 0.25) suggests that this parameter depends on other factors, such as grain size, magnetic domain state, or the degree of aggregation of the magnetic phases.

## 4. Conclusions

The investigated fly ashes differed in mineral composition and magnetic properties depending on the type of coal combusted and the sampling location. Magnetic separation yields of 17.8–54.5 wt.% were not direct indicators of enrichment in iron or ferrimagnetic phases. The magnetic fractions contained both magnetite grains and other spinels, as well as their associations with amorphous Si–Al–Ca glass, which explains the presence of paramagnetic iron in the concentrates. The highest proportion of iron associated with spinels, 61.7–68.4%, was found in magnetic fractions of fly ashes collected from storage silos.

This study demonstrates the applicability of non-contact electrical impedance spectroscopy (EIS) using a measurement coil as a rapid, non-destructive method for discriminating among fly ash samples. The normalized Förster characteristics effectively distinguished the investigated materials, and a single measurement at 131.8 kHz retained discriminative capability. This suggests that complete frequency spectra may in the future be supplemented or partly replaced by rapid single-frequency measurements, without electrode contact with the sample or complex sample preparation.

The combination of Mössbauer spectroscopy, magnetic-susceptibility measurements, and EIS enabled the impedance response to be related to the mineralogical form of iron. The spinel fraction exhibited a very strong correlation with the real component of impedance and magnetic susceptibility measured at low and high frequencies (*r_s_* = 0.964), whereas the imaginary component of impedance showed inverse relationships. These results confirm that impedance parameters depend more strongly on the contribution of magnetically ordered spinel phases than on total iron content. The measured impedance components demonstrate the potential of non-contact EIS as a screening method supporting the assessment of fly ash composition and magnetic properties. Further research involving a larger and more diverse set of samples should validate these relationships and support the development of statistical or machine-learning models for rapid estimation of ferrimagnetic-phase contributions and classification of waste materials.

## Figures and Tables

**Table 1:** List of samples selected for analysis.

Power Plant	Sample Type (Waste Type)	Original Sample Code	Magnetic Fraction Sample Code
B	Fly ash—storage silos	BL1	M-BL1
	Fly ash—storage silos	BL2	M-BL2
	Fly ash—storage silos	BL3	M-BL3
	Fly ash—electrostatic precipitator	BF1	M-BF1
	Fly ash—electrostatic precipitator	BF2	M-BF2
	Fly ash—electrostatic precipitator	BF3	M-BF3
L	Fly ash	LMOS	M-LMOS

**Table 2:** Mass yields of magnetic and non-magnetic fractions obtained by magnetic separation of the investigated samples.

Magnetic Fraction Code	Magnetic Fraction, wt.%	Non-Magnetic Fraction, wt.%	Total, wt.%
M-BL1	20.75	79.25	100.00
M-BL2	17.84	82.16	100.00
M-BL3	18.35	81.65	100.00
M-BF1	30.28	69.72	100.00
M-BF2	54.53	45.47	100.00
M-BF3	44.18	55.82	100.00
M-LMOS=	21.12	78.88	100.00

**Table 3:** Mössbauer hyperfine parameters of the components identified in the spectra of the magnetic fractions of fly ash samples M-BL1, M-BL2, M-BL3. Is—isomer shift; Qs—quadrupole splitting; FWHM—full width at half maximum; H—hyperfine magnetic field; A—relative area fraction of the spectral component.

No.	Is (mm/s)	Qs (mm/s)	FWHM (mm/s)	H (T)	A (%)	Oxydation	Compound
M-BL1							
1	0.37	−0.17	0.29	51.6	9.6	Fe^3+^	Fe_2_O_3_
2	0.29	−0.02	0.46	49.0	28.6	Fe^3+^	Tet-Fe_3_O_4_
3	0.62	−0.02	0.60	45.4	33.0	Fe^2.5+^	Oct-Fe_3_O_4_
4	0.47	0.74	0.60	-	13.2	Fe^3+^	aluminosilicates/silicates
5	0.82	2.17	0.60	-	15.5	Fe^2+^	
M-BL2							
1	0.37	−0.17	0.29	51.4	8.9	Fe^3+^	Fe_2_O_3_
2	0.29	−0.01	0.43	48.8	27.8	Fe^3+^	Tet-Fe_3_O_4_
3	0.61	−0.03	0.60	45.2	36.7	Fe^2.5+^	Oct-Fe_3_O_4_
4	0.51	0.77	0.60	-	12.4	Fe^3+^	aluminosilicates/silicates
5	0.83	2.17	0.60	-	14.2	Fe^2+^	
M-BL3							
1	0.36	−0.18	0.29	51.3	8.4	Fe^3+^	Fe_2_O_3_
2	0.28	−0.02	0.42	48.4	30.5	Fe^3+^	Tet-Fe_3_O_4_
3	0.59	−0.03	0.60	45.2	36.5	Fe^2.5+^	Oct-Fe_3_O_4_
4	0.47	0.78	0.60	-	11.3	Fe^3+^	aluminosilicates/silicates
5	0.79	2.29	0.60	-	11.3	Fe^2+^	

**Table 4:** Mössbauer hyperfine parameters of the components identified in the spectra of the magnetic fractions of fly ash samples M-BF1, M-BF2, M-BF3,M-LMOS. Is—isomer shift; Qs—quadrupole splitting; FWHM—full width at half maximum; H—hyperfine magnetic field; A—relative area fraction of the spectral component.

No.	Is (mm/s)	Qs (mm/s)	FWHM (mm/s)	H (T)	A (%)	Oxydation	Compound
M-BF1							
1	0.36	−0.17	0.60	51.3	22.0	Fe^3+^	Fe_2_O_3_
2	0.22	0.01	0.60	48.0	17.5	Fe^3+^	Tet-Fe_3_O_4_
3	0.60	0.01	0.65	45.3	18.6	Fe^2.5+^	Oct-Fe_3_O_4_
4	0.45	0.79	0.65	-	21.4	Fe^3+^	aluminosilicates/silicates
5	0.80	2.49	0.65	-	20.5	Fe^2+^	
M-BF2							
1	0.37	−0.19	0.29	51.5	19.7	Fe^3+^	Fe_2_O_3_
2	0.29	−0.02	0.55	48.9	25.6	Fe^3+^	Tet-Fe_3_O_4_
3	0.64	0.02	0.62	45.6	24.4	Fe^2.5+^	Oct-Fe_3_O_4_
4	0.19	0.48	0.42	-	14.4	Fe^3+^	aluminosilicates/silicates
5	0.40	1.20	0.62	-	9.0	Fe^3+^	
6	1.07	1.62	0.62	-	9.8	Fe^3+^	
M-BF3							
1	0.37	−0.17	0.62	51.1	21.7	Fe^3+^	Fe_2_O_3_
2	0.29	0.04	0.62	47.0	16.4	Fe^3+^	Tet-Fe_3_O_4_
3	0.58	0.03	0.62	44.0	15.6	Fe^2.5+^	Oct-Fe_3_O_4_
4	0.14	0.34	0.62	-	25.9	Fe^3+^	aluminosilicates/silicates
5	0.64	0.87	0.62	-	10.1	Fe^3+^	
6	0.80	2.30	0.62	-	10.2	Fe^2+^	
M-LMOS							
1	0.37	−0.19	0.28	51.5	11.7	Fe^3+^	Fe_2_O_3_
2	0.27	−0.01	0.53	48.7	21.9	Fe^3+^	Tet-Fe_3_O_4_
3	0.62	−0.02	0.62	45.3	20.9	Fe^2.5+^	Oct-Fe_3_O_4_
4	0.36	0.73	0.62	-	15.8	Fe^3+^	aluminosilicates/silicates
5	1.00	2.58	0.62	-	9.5	Fe^2+^	
6	0.97	1.86	0.62	-	14.7	Fe^2+^	
7	0.84	1.15	0.62	-	6.2	Fe^2+^	

**Table 5:** Normalized results of phase analysis by Mössbauer spectroscopy. The share of iron associated with: *C_he_*—hematite, *C_Al_*—aluminosilicate phase (glassy phase), *C_sp_*—spinel phases, *C_mag_*—magnetically ordered phases (sextets) (hematite and spinel phases), *C_sp_*_/*mag*_—spinel phases among all magnetically ordered phases.

Sample	*C_he_*, %	*C_Al_*, %	*C_sp_*, %	*C_mag_*, %	*C_sp_*_/*mag*_, %
M-BL1	9.61	28.73	61.66	71.27	86.52
M-BL2	8.90	26.60	64.50	73.40	87.87
M-BL3	8.57	23.06	68.37	76.94	88.86
M-BF1	22.00	41.90	36.10	58.10	62.13
M-BF2	19.14	32.26	48.59	67.74	71.74
M-BF3	21.72	46.25	32.03	53.75	59.59
M-LMOS	11.62	45.88	42.50	54.12	78.53

**Table 6:** Magnetic susceptibility at low *χ_lf_* and high *χ_hf_* frequencies and frequency-dependent magnetic susceptibility *χ_fd_*% of the investigated magnetic fractions.

Sample ID/Statistical Parameters	*χ_lf_* [m^3^/kg × 10^−8^]	*χ_hf_* [m^3^/kg × 10^−8^]	*χ_fd_*% [%]
M-BL1	2566.3	2552.2	0.55
M-BL2	3301.3	3268.8	0.98
M-BL3	4396.0	4370.7	0.58
M-BF1	981.3	969.4	1.22
M-BF2	1188.2	1175.8	1.04
M-BF3	978.2	968.8	0.97
M-LMOS	3552.9	3159.0	11.09
Min.	978.2	968.8	0.55
Max.	4396.0	4370.7	1.22
Mean	2235.2	2217.6	0.89
Standard Deviation	1425.8	1418.2	0.27

**Table 7:** Spearman rank correlation coefficient matrix between the magnetic separation parameters and the magnetic properties of the investigated samples.

	*C_he_* %	*C_sp_* %	*C*_*sp*/*mag*_, %	R-Ro/ωLo	ωL/ωLo	*χ_lf_*	*χ_hf_*
*C_sp_*, %	−0.929						
*C*_*sp*/*mag*_, %	−0.964	0.964					
R-Ro/ωLo	−0.857	0.786	0.964				
ωL/ωLo	0.857	−0.786	−0.964	−1.000			
*χ_lf_*	−0.857	0.786	0.964	1.000	−1.000		
*χ_hf_*	−0.929	0.893	0.964	0.964	−0.964	0.964	
*χ_fd_*%	0.220	−0.202	−0.073	0.110	−0.110	0.110	−0.018

## Data Availability

The original contributions presented in this study are included in the article. Further inquiries can be directed to the corresponding author.
